# Impacts of drug resistance mutations on the structural asymmetry of the HIV-2 protease

**DOI:** 10.1186/s12860-020-00290-1

**Published:** 2020-06-23

**Authors:** Pierre Laville, Sandrine Fartek, Natacha Cerisier, Delphine Flatters, Michel Petitjean, Leslie Regad

**Affiliations:** Université de Paris, BFA, UMR 8251, CNRS, ERL U1133, Inserm, F-75013 Paris, France

**Keywords:** HIV-2 protease, Drug-resistance mutations, Structural asymmetry, Structural alphabet

## Abstract

**Background:**

Drug resistance is a severe problem in HIV treatment. HIV protease is a common target for the design of new drugs for treating HIV infection. Previous studies have shown that the crystallographic structures of the HIV-2 protease (PR2) in bound and unbound forms exhibit structural asymmetry that is important for ligand recognition and binding. Here, we investigated the effects of resistance mutations on the structural asymmetry of PR2. Due to the lack of structural data on PR2 mutants, the 3D structures of 30 PR2 mutants of interest have been modeled using an in silico protocol. Structural asymmetry analysis was carried out with an in-house structural-alphabet-based approach.

**Results:**

The systematic comparison of the asymmetry of the wild-type structure and a large number of mutants highlighted crucial residues for PR2 structure and function. In addition, our results revealed structural changes induced by PR2 flexibility or resistance mutations. The analysis of the highlighted structural changes showed that some mutations alter protein stability or inhibitor binding.

**Conclusions:**

This work consists of a structural analysis of the impact of a large number of PR2 resistant mutants based on modeled structures. It suggests three possible resistance mechanisms of PR2, in which structural changes induced by resistance mutations lead to modifications in the dimerization interface, ligand recognition or inhibitor binding.

## Background

Proteases (PRs) are an important therapeutic target in the treatment of HIV-1 and HIV-2 infections because of their indispensable role in Gag and Gag-Pol polyprotein hydrolysis during the viral maturation process [1]. Currently, nine drugs targeting PR for the treatment of HIV-1 have been approved for clinical use by the Food and Drug Administration (FDA), only three of which (darunavir (DRV), saquinavir (SQV), and lopinavir (LPV)) are effective in the treatment of HIV-2 infection [1, 2]. Some studies have shown that the natural resistance of HIV-2 to protease inhibitors (PIs) can be partially explained by substitutions located within the PR2 pocket. These substitutions induce structural changes in pocket residues, modifying pocket properties and the internal interactions between PR2 and PIs, thus altering PI binding [3–10]. Other studies have suggested that substitutions between PR1 and PR2 could modify PI binding by altering the transition between the open and closed forms involved in ligand binding [11, 12].

In addition to the natural resistance of HIV-2 to most HIV-1 PIs, HIV-2 can evolve to achieve drug resistance through the accumulation of mutations on its PR. However, few studies conducted to date provide information about mutations conferring resistance to PIs. Genome sequencing studies of HIV-2 from different HIV-2-infected patients have described the selection of some mutations that confer drug resistance in HIV-2 [7, 13–22]. These resistance mutations were subsequently confirmed using phenotypic susceptibility assays [7, 15–17, 19, 23]. Several studies have shown that a single mutation can confer resistance to one or several PIs, such as V47A, I50V, I54M, I82F, I84V, and L90M [7, 15, 17, 19, 24]. However, some studies have led to contradictory results. For example, the I82F mutation has been observed in the presence of ritonavir (RTV) and indinavir (IDV) in some studies [14, 16, 19], while the I54M mutation has appeared under treatment with amprenavir (APV) [16], nelfinavir (NFV) [14], and IDV [19]. Phenotypic susceptibility assays confirmed that the I82F mutation confers resistance to IDV as well as NFV and LPV [16]. In contrast, Raugi et al. [19] found that this mutation does not increase resistance to LPV but causes hypersusceptibility to both DRV and SQV. In addition, some mutations can appear together to confer high resistance to several PIs [7, 15, 17, 19, 24, 25]. For example, the I54M and I82F mutations confer cross-resistance to all PIs. The V62A and L99F mutations confer cross-resistance to three PIs (IDV, NFV, and LPV) [16]. As no tridimensional (3D) structure of the PR2 mutant is available in the Protein Data Bank (PDB [26]), no structural analysis was performed to study the mechanisms explaining HIV-2 resistance induced by these mutations. However, knowledge of these mechanisms leading to PR2 resistance is important for designing new PR2 inhibitors.

PR2 is a homodimer of 99-residue monomers. The interaction between the two monomers occurs through the Nter, catalytic, and Cter regions. Diverse ligands (peptide substrates with different amino acid sequences and structurally different inhibitors) bind to the PR2 central binding site. The comparison of the unbound and PI-bound PR2 structures showed that the flap region undergoes large structural changes upon ligand binding. In the unbound form, the flap region adopts an open conformation allowing ligand entry. Upon ligand binding, the flap region recloses over the central binding pocket [1].

The analyses of the crystallographic structures of PR2 have shown that the two monomers do not exhibit the same global and local conformations, indicating that in crystallographic structures, PR2 exhibits structural asymmetry [27–32]. This structural asymmetry is translated by slightly different orientation of its two monomers, quantified by a two-fold axis of 178.20° to 179.80° and a root mean square deviation (RMSD) of 0.35 to 1.02 Å [27–30, 33]. The largest deviations between the two monomers are located in the tail, elbow, and flap regions [27–31, 33]. Using a structural-alphabet-based approach, 31% of PR2 positions were identified as structurally asymmetric (i.e., exhibiting different local conformations in their two chains), among which 75% were located outside of the PI-binding pocket [31, 33]. In a recent study, we explored the structural asymmetry of unbound PR [33]. Its two monomers exhibit a *Cα-RMSD* value of 0.53 Å, which is smaller than that computed on the bound PR2 [27–30]. Our structural-alphabet-based approach highlighted that 35% of unbound PR2 positions are asymmetric. In the unbound and bound structures, the asymmetric positions are distributed throughout the structure, particularly in the interface region and in the flap, fulcrum, elbow, and α-helix regions and the binding site [31, 33]. Thus, the crystallographic PR2 structure exhibits structural asymmetry in its backbone, and this property is also found in the unbound structure. These results highlighted the asymmetric properties of the crystallographic structures of PR2, which are not caused by ligand binding alone. Indeed, proteins are dynamic objects that adjust the positions of their atoms to respond to different events, such as partner binding. In the case of the crystallographic PR2 structures, the structural asymmetry results from crystal packing [27, 28, 30, 33], protein dimerization [31], and ligand binding [28, 29, 31]. Different studies have differentiated the PR2 asymmetry induced by ligand binding that is important for ligand recognition and binding [28, 29, 31] to structural asymmetry corresponding to an intrinsic factor allowing the structural deformation of the target [34–36]. For example, Mulichak et al. [28] showed that the binding of a peptidic inhibitor in the PR2 specifically induces a move of the region 79–82 of chain B allowing inhibitor binding.

In this work, we explored the structural effects of some drug resistance mutations of PR2 by comparing the structural asymmetry of the wild-type and drug-resistant mutants of PR2. The studied PR2 drug-resistant mutants harbored one, two or three mutations. As no structural data are available for these PR2 mutants, we constructed their 3D structures using molecular modeling as in [10]. We then detected structural asymmetry (i.e., positions exhibiting different local conformations in the two PR2 chains) in the wild-type and mutant structures using our structural-alphabet-based approach [31]. The comparison of the structural asymmetry of wild-type and mutant structures highlighted three possible mechanisms that could explain PR2 resistance to PIs.

## Results

### Quantification of PR2 structural deformation induced by drug resistance mutations

**Fig. 1:**
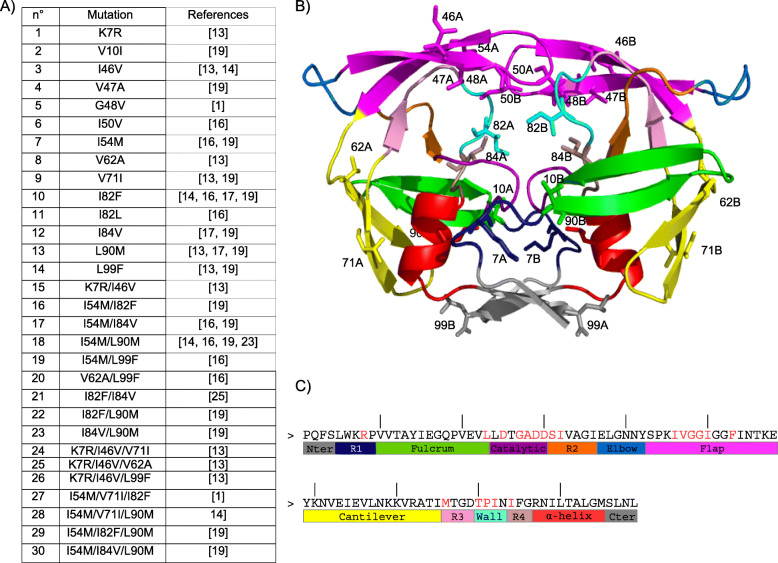
Presentation of drug-resistant mutants. **a** List of the 30 drug-resistant mutants with their different mutations. **b** Localization of the mutations observed in the 30 drug-resistant mutants onto the 3D structure of wild-type PR2. PR2 is displayed in cartoon mode and colored according to the 13 extracted PR2 regions defined in [32, 37, 38]. Each PR2 region is colored as follows: the Nter and Cter regions in grey, the R1 region in dark blue, the fulcrum region in green, the catalytic region in purple, the R2 region in orange, the elbow region in blue, the flap region in magenta, the cantilever region in yellow, the R3 region in pink, the wall region in cyan, the R4 region in brown, and the α-helix region in red. Positions, where drug-resistance mutations occur, are displayed in stick mode. **c** Limits of the 13 structural and functional regions extracted from PR2. The 20 pocket residues were highlighted in red

We focused on a set of 30 drug-resistant mutants containing from one to three mutations (Fig. 1a). The 3D structure of each mutant was built using FoldX software [39] with five replications (see Methods), resulting in a set of 150 mutant models. To compare the mutant and wild-type structures, we computed the all-atom RMSD, denoted as *RMSD*_*aa*_, between the mutant structure and the three minimized wild-type structures (3EBZ_mini_). The five structures of each mutant could exhibit large *RMSD*_*aa*_ (i.e., different conformations) (Additional file 1: Figure S1). As expected, single mutants (i.e., those with only one mutation) exhibited a smaller average *RMSD*_*aa*_ than the other mutants, indicating that introducing a single mutation induces less structural change than introducing several mutations (Additional file 1: Figure S1). In addition, there was a link between the number of mutations and structural diversity in terms of *RMSD*_*aa*_. Indeed, the three types of mutants (single, double and triple) did not exhibit the same variability in terms of *RMSD*_*aa*_ (Bartlett-test *p*-value = 2.10^− 11^): triple mutants showed more conserved structures than the other types of mutants. Thus, compared to single or double mutants, the insertion of three mutations induced more structural diversity relative to the wild-type structure, but the five modeled mutant structures were more similar to each other.

### Detection of structural asymmetry in the wild-type and mutant structures of PR2

To explore the structural effects of the studied drug resistance mutations, we compared the structural asymmetry in the three 3EBZ_mini_ structures and the 150 modeled mutant structures using an approach based on a structural alphabet [31].

### Characterization of structural asymmetry in the three 3EBZ_mini_ structures

**Fig. 2:**
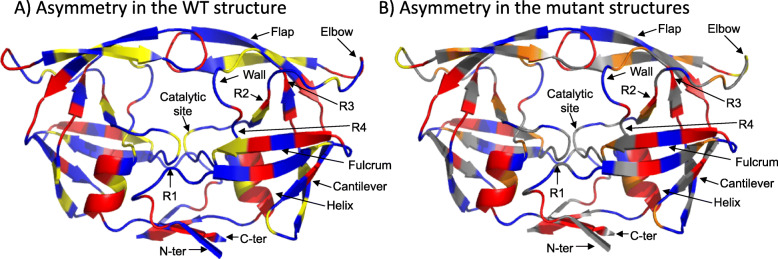
Localization of asymmetric and symmetric positions in the wild-type and mutant structure. **a** 3EBZ_mini_ structure colored according to residue asymmetric behavior in the three wild-type structures: the 51 residues that are symmetric in the three 3EBZ_mini_ structures are colored blue, the 27 residues asymmetric in the three 3EBZ_mini_ structures are colored red, and the 18 residues exhibiting different asymmetric statuses in the three 3EBZ_mini_ structures are colored yellow. **b** 3EBZ_mini_ structure colored according to residue asymmetric status in the mutant set: the 25 residues that are symmetric in the 150 mutants are blue, the 25 residues that are overrepresented in terms of asymmetry in the mutant set and are asymmetric in the three 3EBZ_mini_ structures are red, the 11 residues that are overrepresented in terms of asymmetry in the mutant set and are symmetric in the three 3EBZ_mini_ structures are orange, and the 2 residues that are asymmetric in the three 3EBZ_mini_ structures and are not overrepresented in the mutant set are yellow. Additional file 2: Figure S2 lists the positions of each type in the wild-type and mutant sets and their distributions in the 13 regions

We determined the structural symmetric and asymmetric positions in the three 3EBZ_mini_ structures and their location in the 13 PR2 regions defined in  [32, 37, 38] (Additional file 2: Figure S2). Half of the positions (53% of positions) were detected as symmetric (i.e., showing similar local conformations in the two chains in the three 3EBZ_mini_ structures). The entire PR2 structure was sampled, particularly the fulcrum, flap, and cantilever regions (Fig. 2a). Thus, the conserved local conformation in both monomers at these positions is important for PR2, particularly for pocket residues 28, 30, 53, 81 and 82, which could be important for ligand binding. The three 3EBZ_mini_ structures contained between 34 and 38 asymmetric positions, with 28% of the positions showing asymmetry in the three 3EBZ_mini_ structures. These positions were located throughout the structure, particularly in the α-helix and cantilever regions (Fig. 2a). This asymmetry conservation suggests an important role of these positions, particularly for the elbow and flap positions (positions 40–42, 50, 51, 58), which could be important for PR2 deformation.

A total of 18 positions did not exhibit the same asymmetry status in the three 3EBZ_mini_ structures (Fig. 2a). These 18 positions were not more flexible (in terms of B-factor value) or more accessible (in terms of accessible surface area (ASA) values) residues relative to other positions (Kruskal-Wallis test *p*-values = 0.25 and 0.46, Additional files 3 and 4: Figures S3 and S4). This suggests that the asymmetric variability of these positions does not result from the intra-flexibility of PR2. However, we have previously shown that these 18 positions exhibit different conformations in the 18 available structures of PR2 complexed with different ligands [32]. Thus, these positions could modify their local conformation to adapt to different ligands.

### Characterization of structural asymmetry in the PR2 mutant set

On average, a mutant structure contains 31.8 ± 4.4 asymmetric positions, which is close to the number of asymmetric positions in the three 3EBZ_mini_ structures. The five structures of a given mutant do not always have the same number of asymmetric positions and can exhibit few common asymmetric positions, such as the G48V mutation (m5); see Additional file 5: Figure S5. The variability of the number of asymmetric positions per mutant does not depend on the number of mutations (Bartlett test *p*-value = 0.14). As expected, a link was found between variability in terms of *RMSD*_*aa*_ and both (i) the variability in terms of the number of asymmetric positions (Pearson correlation coefficient = 0.73) and (ii) the number of common asymmetric positions between the five mutant structures (Pearson coefficient between the standard deviation of the *RMSD*_*aa*_ value for the 5 mutant conformations and the number of common asymmetric positions = − 0.65). Thus, the mutants exhibiting greater diversity in terms of *RMSD*_*aa*_ corresponded to the mutants showing five structures exhibiting different numbers of asymmetric positions with few common asymmetric positions.

To characterize the structural asymmetry in the mutant set, we then computed the asymmetry occurrence (*AO*) for each position (i.e., the number of mutant structures exhibiting asymmetry for a considered position). A total of 26% of the positions that were symmetric in all mutant structures were also symmetric in the three 3EBZ_mini_ structures. This indicates that the resistance mutations do not affect the structural symmetry of these positions, particularly the mutations occurring at some of these symmetric positions (K7R, V10I, V71I, I82F, and I82L). These positions were frequent in the fulcrum, flap, and cantilever regions and absent in the Nter, Cter, elbow, R3, and R4 regions (Fig. 2b). Five of them (28, 30, 53, 81, 82) were located in the binding site, confirming the important role of these positions in ligand binding.

In the mutant set, 36 positions correspond to overrepresented asymmetric positions and were denoted as *OR*_*asym*_ positions. Their asymmetry does not arise at random. These *OR*_*asym*_ positions are located throughout the structure except in the R1 and catalytic regions (Fig. 2b). These positions did not consist of more flexible (in terms of B-factor) or exposed (in terms of ASA) residues on average than other asymmetric positions (T-test *p*-values = 0.40 and 0.53, respectively, additional files 3 and 4: Figures S3 and S4). Seventy percent of the *OR*_*asym*_ positions were also asymmetric in the three 3EBZ_mini_ structures. These positions were particularly common in the α-helix, flap, cantilever, and fulcrum regions (Fig. 2b). Thus, the studied drug resistance mutations do not modify the structural asymmetry of these positions, reinforcing the important role of the structural asymmetry of these positions. These overrepresented asymmetric positions could be important for PR2 structure or activity, particularly the four residues belonging to the dimerization region (4, 5, 97, and 98) and the five pocket residues (23, 32, 47, 50, 80).

The remaining 11 *OR*_*asym*_ positions were symmetric in the three 3EBZ_mini_ structures. In addition, two positions (40 and 41) were asymmetric in the three 3EBZ_mini_ structures and were not overrepresented in the mutant set. Among these 13 positions, four were close (44 and 80) or corresponded (47 and 90) to mutated positions. Thus, these drug resistance mutations could be responsible for the changes in asymmetry at these mutated positions and their nearby residues, and they could modify the asymmetry of more distant residues.

### Link between drug resistance mutations and changes in asymmetry occurring in mutants

**Fig. 3:**
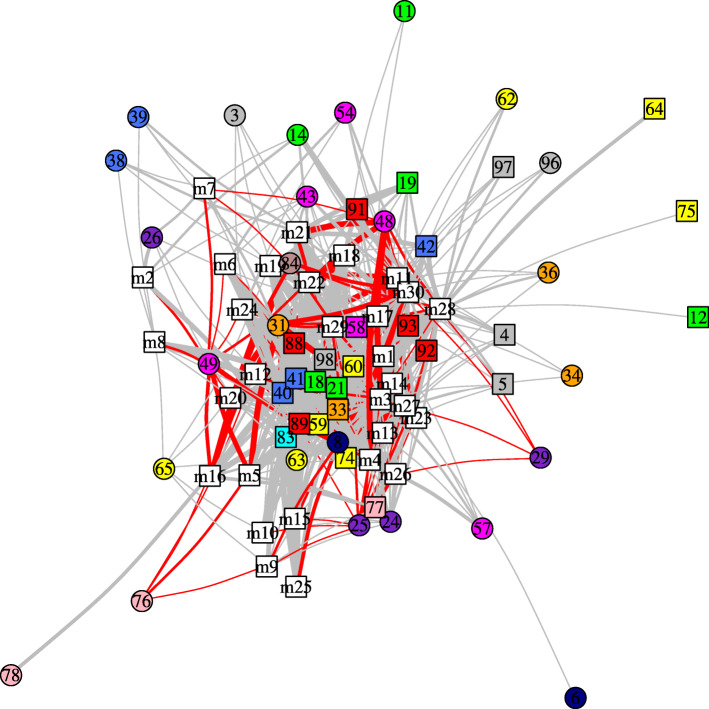
Network summarizing the link between the 30 drug-resistant mutants and changes in asymmetry. In this network, white square nodes correspond to mutants, and colored nodes (square and circle) correspond to positions where a change in asymmetry occurs. Positions are colored according to the 13 regions extracted from the PR2 structure. See Fig. 1b for the region color legend. The shape of position nodes indicates the type of change in asymmetry occurring at the studied position: the circular nodes indicate asymmetry and the square nodes indicate the loss of asymmetry. This network connects a mutant to a position if the position presents a change in asymmetry in at least one of its five structures. The edge thickness is proportional to the number of mutant structures exhibiting the change (ranging from 1 to 5). The edges are colored according to whether the change in asymmetry occurred at a position located in the binding pocket (in red) or outside of the binding pocket (in gray)

For each mutant, we determined how many of its five structures exhibited a change in asymmetry at each position relative to the three wild-type structures. The number of changes in asymmetry observed for each mutant varied from 6 (mutant m2) to 36 (mutant m5) and did not depend on the number of mutations (*P*-value of the Kruskal-Wallis test = 0.55). Figure 3 presents the network connecting a mutant with its asymmetric positions. We observed that some changes in asymmetry occurred at positions connected with many mutations (which correspond to central nodes in the network), while others were connected to few mutations (which correspond to external nodes in the network, Fig. 3). For example, changes in asymmetry at positions 40, 41, 33, 18, and 98 were observed in more than 20 mutants (Fig. 3). These changes in asymmetry were not specific to certain mutations, suggesting that they were not induced by mutations. In contrast, structural backbone asymmetry at positions 6 and 78 was observed only in mutants I54M/I84V (m17) and K7R/I46V/L99F (m26), respectively, while such asymmetry at position 62 was observed in mutants I84V (m12), G48V (m5), and I84V/L90M (m23). The loss of structural asymmetry at positions 12, 64, and 75 was only observed in mutant G48V (m5), but the five structures of these mutants did not exhibit this loss.

**Fig. 4:**
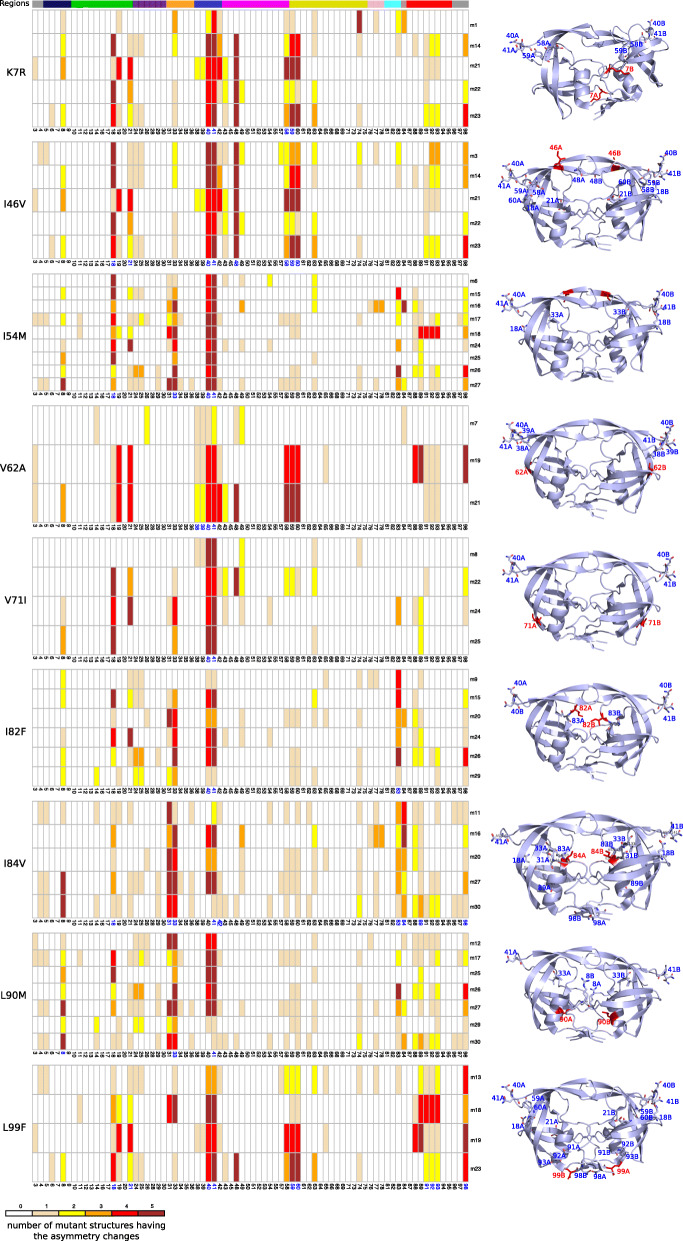
Detection of changes in asymmetry in mutant structures. For this analysis, we selected all structures exhibiting the K7R, I46V, I54M, V62A, V71I, I82F, I84V, L90M, or L99F mutation. **a** presents matrices summarizing the positions exhibiting changes in asymmetry for the nine selected mutations. The matrix rows present mutant and matrix columns corresponding to PR2 positions. A matrix cell indicates the number of mutant structures (from 0 to 5) exhibiting changes in asymmetry at a given position. Positions in blue exhibit changes in asymmetry in all mutants. PR2 regions are presented at the top of the figure and are colored according to the color code of Fig. 1. **b** Localization of positions exhibiting changes in asymmetry in the PR2 structures. Proteins are displayed in cartoon mode and colored blue. Mutations are displayed with sticks and colored red. Positions exhibiting changes in asymmetry in the mutant structures relative to the wild-type structure are displayed in stick mode. Only positions exhibiting changes in asymmetry in at least one structure of all mutants harboring the studied mutations are indicated in the 3D structure of PR2

To highlight the changes in asymmetry that are putatively induced by resistance mutations, we studied the conservation and location of structural changes in all structures containing at least one of the nine mutations observed in multiple mutants (K7R, I46V, I54M, V62A, V71I, I82F, I84V, L90M, and L99F); see Fig. 4. We noted that some changes in asymmetry occurred in all mutants exhibiting a given mutation. For example, a structural change at position 83 was observed in all mutants exhibiting the I82F or I84V mutation. The location of conserved structural changes in the PR2 structures allowed us to differentiate two types of changes in asymmetry: those occurring far from a mutated residue and those occurring close to a mutated residue, which were putatively induced by mutation.

### Asymmetric changes occurring far from mutated residues and, thus, putatively not related to mutations

Figure 4 shows that the nonspecific changes in asymmetry occurred at positions 40 and 41 in most mutants, but they were located far from mutated residues. As previously assumed, the high frequency of these two changes in asymmetry suggests that they are not induced by a mutation. This was confirmed by the fact that they occurred at two exposed residues located among the elbow residues (Additional file 4: Figure S4). Thus, the loss of asymmetry observed at positions 40 and 41 could be induced by residue flexibility. In the mutants with K7R, I46V, I54M, I84V, or L99F mutations, changes in asymmetry at positions 40 and 41 were accompanied by the loss of asymmetry at positions 18, 21, 58, 59, and 60 (Fig. 4). As these positions are located close to flexible residues 40 and 41, we suggest that these changes in asymmetry could be induced by the changes in asymmetry at positions 40 and 41. The nine mutants with the I54M mutation also presented a loss of asymmetry at position 33, which was also observed in all mutants exhibiting either the I84V or L90M mutation. Although this change in asymmetry was specific to certain mutations, it was not located close to the mutated residues (Fig. 4). The seven mutants containing the L90M mutation exhibited asymmetry at position 8, which was located far from the mutated residue 90 (Fig. 4). Thus, it is difficult to reach a conclusion regarding the link between the changes in asymmetry that occurred at position 33 or 8 and mutations I54M and L90M.

### Asymmetric changes close to mutated residues putatively related to mutations

Figure 4 shows that the three mutants with the V62A mutation exhibited asymmetry at positions 38 and 39 and loss of asymmetry at positions 40 and 41. Mutated residue 62 was close to residue 38, which was close to residues 39–41 (Fig. 4). Thus, the V62A mutation could be responsible for the changes in asymmetry at positions 38–41. However, the loss of asymmetry at positions 40 and 41 was highly recurrent in the mutant set, whereas the asymmetry at positions 38 and 39 was specific to V62A. Thus, it is difficult to conclude that the V62A mutation induces changes in asymmetry at positions 38 and 39.

Some changes in asymmetry occurred at residues involved in the PI-binding pocket. For example, all mutants containing the I46V (or I84V) mutation exhibited asymmetry at pocket residue 48 (or 31 and 84). As residues 48 and 84 establish hydrophobic contacts with PIs (Additional file 6: Figure S6), we concluded that the I46V and I84V mutations could induce structural backbone changes at positions 31, 48, and 84 that could modify structural asymmetry and PI interactions.

Other changes in asymmetry occurred at residues that were located outside of the binding site but were important for its conformation, such as residues 33, 83, and 89 [10, 32]. Figure 4 shows that the structures of all mutants with I82F, I82L, or I84V mutations exhibited a loss of asymmetry at these important positions. Thus, these changes in asymmetry could be a consequence of structural changes induced by mutations at positions 82 and 84, which could modify the conformation of the binding pocket and, thus, indirectly alter PI binding.

Concerning the L99F mutation, we observed that the four mutants containing this mutation exhibited changes in asymmetry at positions 91, 92, 93, and 98. Residues 91, 92, 93, and 98 are involved in the interface between the two monomers: residues 91 and 92 establish non bonded contacts at the interface, and residue 98 establishes hydrogen bonds with residues 2 and 96 of the second monomer (Additional file 7: Figure S7). Thus, the L99F mutation could induce structural asymmetry at interface residues that could have an impact on the PR2 interface and modify the stability of the dimer.

## Discussion

In this study, we explored the impact of resistance mutations arising in the PR2 target on its backbone asymmetry to obtain information about the structural effects of these mutations. Several studies have shown that although PR2 is a homodimer, its crystallographic structures exhibit structural asymmetry that is important in the mechanism of PI recognition and binding [10, 27–30, 33]. In this work, we compared the local structural asymmetry of a wild-type PR2 structure and 30 resistant mutants. As no structure of the PR2 mutant was available in the PDB, we modeled the structure of the 30 studied mutants using the protocol developed in our previous study [10] based on FoldX software [39] and an energy minimization step. As FoldX software is based on a side-chain rotamer library, we built five structures per mutant to consider the different rotamers associated with each amino acid. The comparison between the wild-type and mutant structures confirmed the importance of generating several structures per mutant.

In the first step of this study, we analyzed the structural asymmetry in three minimized wild-type structures of PR2 and a set of 150 modeled mutant structures. Considering a large set of PR2 structures (wild-type and mutants) at the same time allowed us to extract information about the role of particular residues. Our results highlighted 25 residues that are symmetric in the wild-type and mutant structures i.e. presenting the same local conformation in the two monomers. Eight of these residues (17, 28, 30, 53, 68, 81, 82, 87) were previously detected as symmetric in a set of 19 structures of wild-type PR2 available in the PDB [31], in which they exhibited the same conformation [32]. This conservation of symmetric status highlights the important role of the symmetry of these residues for PR2. As residues 28, 30, 53, 81, and 82 are located in the PR2 pocket and residue 87 establishes interactions with pocket residues 28–29 [10, 30, 32], we concluded that the conserved conformation of these residues in the two chains is important for the binding site conformation and ligand binding. In contrast, our results identified 25 residues that were characterized as asymmetric in the three minimized wild-type structures and overrepresented in terms of asymmetry in the mutant structures. Twelve of these 25 asymmetric positions (18, 42, 50, 51, 59, 60, 64, 75, 77, 83, 92, and 93) were previously detected as overrepresented in terms of asymmetry in the 19 PR2 structures available in the PDB [31]. These results confirm that the backbone asymmetry of these residues is important for PR2 structure and function [31]. In addition, residues 18, 42, 59, and 75 are involved in the H-bond network with residues of the elbow, which is an important region for the flexibility of PR2 [32]. Thus, the structural asymmetry of these residues seems to be important for the transition between the PR2 open and closed forms. Residues 50 and 51 are involved in the interface of the two monomers, suggesting that the backbone asymmetry of these residues is induced by dimerization. Residue 83 has been previously shown to be important for the binding site conformation [28, 30, 32]. Thus, the structural asymmetry of residue 83 could be important for PI recognition and binding. All these results confirm that backbone asymmetry is an intrinsic property of PR2 that is involved in its flexibility and ligand recognition and binding. They also confirm the interest in mining several structures associated with a target to offer valuable insight into target structure, flexibility and interaction mechanisms [40–42].

We showed that wild-type and mutant PR2 structures exhibit different asymmetric statuses at some positions. We distinguished changes in asymmetry between wild-type and mutant structures occurring at the same positions in many mutants. Some of these changes in asymmetry occurred at flexible residues and sites located far from mutated residues, such as residues at 18, 40, and 41. Thus, these changes in asymmetry were putatively induced by PR2 intra-flexibility. On the other hand, some changes in asymmetry occurred in some mutants at positions close to the mutated residues, suggesting that these changes in asymmetry resulted from structural changes induced by mutations. By analyzing the locations of these changes in backbone asymmetry, our results suggested different putative mechanisms of resistance, as observed in PR1 [43]. First, we proposed that resistance mutations could induce structural changes at the interface of the two monomers. For example, our results suggest that the L99F mutation might induce changes in asymmetry at interface positions 91, 92, 93, and 98, which could modify the PR2 interface and alter its stability. This deformation of the dimer interface induced by resistance mutation was previously observed in PR1 mutants with an L24I, F53L or I50V mutation [44, 45]. Liu et al. [44] showed that the L24I mutation induces structural changes in PR1 that modify the contacts between the two Cter regions formed by residues 95–99, which explains the reduced stability in urea and the increased dissociation of the dimer. Second, our results suggest that some resistance mutations could directly modify PI binding. For example, we showed that mutations I46V and I84V could induce structural changes at pocket residues 31, 48, and 84, where the last two residues interact with PIs through hydrophobic contacts. Thus, these structural changes located in the PI-binding pocket induced by mutations could directly alter PI binding. This resistance mechanism has been previously observed in some PR1 mutants. For example, Tie et al. [6] showed that mutation I84V induces structural changes in PR1 resulting in the loss of two van der Waals contacts between residue 84 and DRV. The third putative resistance mechanism corresponds to induced structural changes outside of the binding pocket but within residues important for the binding site conformation. Indeed, we showed that the I84V mutation could induce changes in asymmetry at positions 33, 83, and 89 that are involved in the hydrogen-bond network with pocket residues [32]. Thus, the I84V mutation could also alter the PI-binding pocket by modifying pocket properties through structural changes at positions 33, 83, and 89.

One possible explanation for the observed structural asymmetry that we did not consider in this study is the error or packing in the crystallographic structure. We previously showed that 24 positions were involved in crystal packing in the PR2 structure complexed with DRV (PDB code 3EBZ) [37]. In addition, we previously highlighted some structural asymmetry linked to crystal packing in unbound PR2, for example, at positions 3 and 18 [33]. This suggests that the detected conservation of the asymmetry of some positions results from crystal packing. However, in this study, structural asymmetry was extracted from structures (crystallographic structures and models) that were energetically minimized. This process enables the removal of crystal packing and contact with no biological relevance in the PR2 dimer. Indeed, we have previously shown that minimized structures exhibit fewer structural asymmetric positions than crystallographic structures [33]. Thus, using energetically minimized structures decreases the impact of crystal errors and packing on structural asymmetry. One solution to remove this source of asymmetry could be to extract structural asymmetry from structure models generated during molecular dynamics simulations. In addition, the analysis of asymmetry during molecular dynamics simulations could provide information about the link between structural asymmetry and PR2 deformations induced by these substitutions and facilitate a detailed understanding of the role of these substitutions. One limitation of our study is that we focused on the structural changes occurring in the backbone of PR2 without considering side-chain deformations. Even though our results suggest the existence of some structural deformations induced by mutations that could lead to PR2 resistance to PIs, we may have missed some mechanisms. For example, we observed that all mutants with the L90M mutation exhibited a change in asymmetry at position 8. As positions 8 and 90 were distant from each other in the PR2 structure, we concluded that this structural change was not induced by this mutation. However, one possibility is that the L90M mutation could have induced structural changes in some side chains that were not detected by our approach. These structural changes could spread within the structure to lead to the deformation of the backbone of pocket residue 8. Thus, to better understand the resistance of PR2 induced by some mutations, it would be interesting to consider the deformations occurring in side chains in the future. Another limitation of our study is that we considered only one template during the mutant structure-modeling step: wild-type PR2 complexed with DRV (PDB code 3EBZ [46]). However, not all studied mutants are resistant to DRV. For example, Raugi et al. [19] showed that the L90M mutant is resistant to SQV and exhibits only a weak decrease in susceptibility to DRV. Thus, the use of a single template complexed with only one PI could explain why, for some mutants, we did not identify significant structural changes. It would be interesting to consider the complete resistance profile of each mutant. This will require the construction of mutant structures complexed with all PIs and knowledge of the resistance profile of each mutant. However, the resistance profile is particularly difficult to obtain for all mutants because there are few studies that have tested the effect of PIs against HIV-2 mutants using enzymatic or phenotypic susceptibility assays [9, 16, 17, 19, 23] and some of these studies have led to opposing results [1].

## Conclusions

In this paper, we studied the structural impact of resistance mutations occurring in PR2, an important therapeutic target in HIV-2 infection treatment. More specifically, we explored the effect of resistance mutations on structural backbone asymmetry, a property involved in dimerization, ligand recognition and binding. To do so, we detected the differences in terms of structural asymmetry between the wild-type and 30 modeled mutant structures. Studying a large set of mutant structures at the same time allowed us to confirm the functional and structural roles of some PR2 residues, such as residues 28, 30, 53, 81, 82, and 87, that have been identified as important for the binding site conformation and ligand binding.

In addition, the comparison between the structural asymmetry of wild-type and mutant structures allowed the detection of some structural changes that could be induced by either PR2 flexibility or the studied resistance mutations. The analysis of the latter type of structural changes revealed three possible resistance mechanisms of PR2 that could occur together. First, we observed that resistance mutations could modify the PR2 interface and its stability, such as mutation L99F that induces structural changes at interface positions 91, 92, 93 and 98. The second mechanism involves the alteration of the PI interaction directly induced by mutations, such as the I46V and I84V mutations that induce structural changes at pocket residues. The third resistance mechanism corresponds to indirect modifications of PI binding. We noted that the I84V mutation could modify the PI-binding site by inducing structural changes at residues important for the maintenance of the conformation of the PI-pocket binding site.

In conclusion, this study was a structural analysis of the impact of a large number of PR2 resistance mutations using modeled mutant structures. Our results provide a better understanding of the structural effects of mutations of PR2 and, thus, of PR2 resistance to PIs.

## Methods

### Presentation of the wild-type PR2 structure

To model the PR2 mutant structures, we used the crystallographic structure of PR2 in complex with DRV (PDB code: 3EBZ [46]). We chose this structure because it is the only available structure of PR2 complexed with one of the three FDA-recommended drugs for the treatment of HIV-2 infection (DRV, LPV, and SQV). This structure shows a good resolution of 1.20 Å.

### Extraction of structural and functional regions of PR2

We considered thirteen regions extracted from each PR2 monomer as in [32, 37, 38] (Fig. 1b and c). The binding pocket (20 residues per chain) was determined using the limits presented in [32] (Fig. 1c).

### Location of flexible and rigid residues of PR2

The flexibility of PR2 residues was quantified using B-factor values found in the PDB file that measure the atomic displacement factor of residues [47]. From the 3EBZ structure, we extracted the B-factor values of each atom. For each residue, the average B-factor value of its atoms was then calculated. The higher the average B-factor value of a residue is, the more flexible the residue is.

### Location of accessible and buried residues of PR2

The ASA value of protein residues is usually used to differentiate surface-exposed, important for protein interactions, and buried residues, important for protein stability [48]. According to [49], the ASA value is defined as the area of the surface swept out by the center of a probe sphere rolling over a molecule. To compute the ASA value of PR2 residues, NACCESS software [49] was run using 3EBZ structure and a radius probe sphere of 1.4 Å. The higher the ASA value of a residue is, the more accessible the residue is.

### Modeling of mutant PR2 structures

We selected 30 drug-resistant mutants of PR2 containing one (14 single mutants), two (9 double mutants), or three mutations (7 triple mutants, Fig. 1a) from the literature. Figure 1b indicates the location of the mutations in the selected PR2-drug resistant mutants according to the wild-type structure (PDB code: 3EBZ). Six of them (46, 47, 48, 50, 82, and 84) were located in the binding pocket.

As the 3D structure of these mutants was not available in the PDB, we modeled their 3D structure using an in silico protocol based on the FoldX suite [39], as we described previously [10]. To do so, we applied the following protocol to the PR2 crystallographic structure in complex with DRV (PDB code: 3EBZ). First, we prepared the protein structure by removing the DRV ligand, metal atoms, and water molecules. Second, we applied the *RepairPDB* command of the FoldX suite to reduce the energy content of the structure. Third, we performed in silico mutagenesis using the *BuildModel* command of the FoldX suite. This command first introduces one or several mutations in the two chains of the wild-type structure using a side-chain rotamer library. Second, it performs an optimization of side chain of amino acids in the vicinity of the mutated residue(s). Each model was generated with five replications, and other options were set to the defaults. At the end of the mutagenesis step, five structures were modeled per mutant. This resulted in a set of 150 mutant structures for the 30 selected drug-resistant mutants. Fourth, we applied an energetic minimization protocol to the 150 modeled mutant structures using the protocol developed in [12]. The monoprotonated state was assigned to the oxygen atom OD2 of Asp25 in chain B using PROPKA software [50]. The system was solvated in a truncated octahedron box of explicit solvent (TIP3P water model) with a 12.0 Å buffer in each dimension. An appropriate number of chloride ions were added to produce a neutral charge in the system. Protein and water molecules were described using the force field AMBER ff99SB [51]. A two-step energy minimization was carried out in GROMACS [52] using a combination of steepest descent and conjugate gradient algorithms of roughly 1000 and 2000 iterations. Water molecules and counterions were relaxed through a first step energy minimization, using a position harmonic restraining force of 100 kcal.mol^-1^ Å^-2^ on the heavy atoms of the protein. A second step of energy minimization was performed by removing restraints on protein atoms. The particle mesh Ewald (PME) method was adopted to treat the long-range electrostatic interactions [53, 54]. The cutoff distances for the long-range electrostatic and van der Waals interactions were set to 10.0 Å.

We also applied the minimization protocol to the 3EBZ crystallographic structure with three replications, resulting in three minimized structures of wild-type PR2 referred to as the 3EBZ_mini1_, 3EBZ_mini2_, and 3EBZ_mini3_ structures.

### Comparison of wild-type and mutant structures

We computed the RMSD values between the 150 mutant structures and the three 3EBZ_mini_ structures. The mutant structures were optionally superposed onto the 3EBZ_mini_ structures using PyMOL software [55]. The all-atom RMSD values, denoted as *RMSD*_*aa*_, were computed between the superimposed structures using PyMOL software [55].

To analyze the impact of the number of mutations introduced in the mutants on the structural variability between the mutants and the wild-type structures, we compared the average *RMSD*_*aa*_ values of the single, double, and triple mutants using a Kruskal-Wallis test and pairwise Wilcoxon tests. We also compared the variability in terms of the *RMSD*_*aa*_ values of single, double, and triple mutants using a Bartlett test and pairwise Fisher tests.

### Analysis of the structural asymmetry of PR2

*Detection of structural asymmetry in PR2 structures.*

**Fig. 5:**
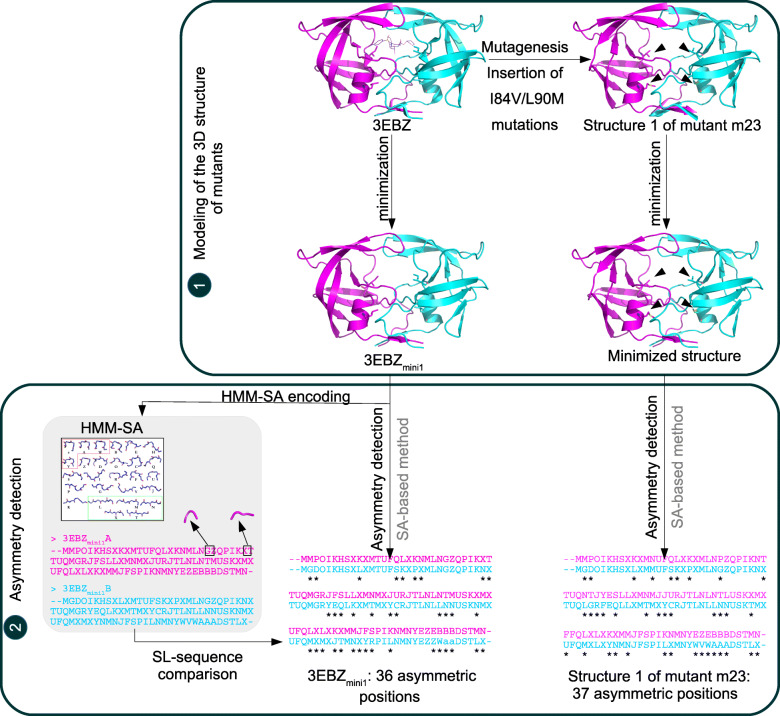
Protocol used to compare the structural asymmetry of the wild-type and mutant structures of PR2. Step 1: Modeling of the 3D structure of PR2 mutants. First, the mutation is introduced into the wild-type structure of PR2 (PDB code: 3EBZ) using FoldX software [39] with five replications. Then, an energetic minimization step is applied to the wild-type and modeled mutant structures using Gromacs software [52]. Wild-type and mutant structures are presented in cartoon mode and colored according to their two chains: chain A is colored magenta, and chain B is colored cyan. Mutated residues are presented in stick mode and are indicated with black arrows. Step 2: Detection of structural asymmetry in the wild-type and mutant structures of PR2 using a protocol based on the HMM-SA structural alphabet. The HMM-SA structural alphabet [56] is a library of 27 protein prototypes of 4 residues classified according to their geometry. First, HMM-SA is used to simplify each PR2 chain of 99 residues into a sequence of 96 structural letters, in which each structural letter corresponds to the geometry of a 4-Cα fragment (i.e., representing the local conformation of each residue). Then, the structural letter for each position in the two sequences is compared to localize (i) symmetric positions that correspond to positions exhibiting the same local conformation (=structural letter) in the two chains of the dimer and (ii) asymmetric positions that correspond to positions exhibiting different local conformations (=structural letters) in the two chains of the dimer. To quantify the structural asymmetry in PR2 structures, the number of asymmetric positions in the dimer is finally counted

The detection of structural asymmetry in a dimer corresponded to the identification of positions that exhibit different backbone conformations in the two chains. In this study, we extracted structural asymmetry from the three minimized wild-type structures of PR2 and the 150 modeled mutant structures using the structural alphabet-based approach that we previously developed [31]. This protocol, presented in Fig. 5, is based on the HMM-SA structural alphabet (Hidden Markov Model - Structural Alphabet), which is a library of 27 prototypes of 4-Cα residues classified according to their geometry, assigned structural letters and labeled [a, A-Z] [56]. First, HMM-SA was used to simplify the 3D structure of the two chains of *p* residues in two sequences of (*p-3*) structural letters overlapping by 3 residues, where each structural letter corresponded to the geometry of a 4-Cα fragment. Each structural letter was assigned to the third residue of the 4-residue fragment. As the fragments overlapped by 3 residues, no structural letter was assigned to the first, second, and last residues. Second, the structural letters of each position in both chains were compared. A position has two possible asymmetry profiles: either the position is asymmetric (i.e., exhibiting different local conformations) (=structural letters) in the two chains, or it is symmetric (i.e., exhibiting the same local conformation) (=structural letter) in the two chains.

### Quantification of structural asymmetry

We first counted the number of asymmetric positions in the three minimized wild-type and 150 mutant structures, as described in [31]. To quantify the structural asymmetry in the mutant structure set, we determined the occurrence of asymmetry (*AO*) at each position, *i* (i.e., the number of mutant structures exhibiting asymmetry at position *i*). The statistical significance of the *AO* value of position *i* was determined using the overrepresentation *p*-value, *p*_*AO*_. The *p*_*AO*_ of position *i* was computed by comparing its observed (*AO(i)*) and expected (*AO*^*random*^*(i)*) occurrences. The *AO*^*random*^ occurrence of position *i* was computed in a random set generated with 150 random binary sequences of 96 positions, where each random sequence was associated with a mutant structure. In each random binary sequence, the 0 and 1 values represent the symmetric and asymmetric positions, respectively. The number of asymmetric positions in the random sequence, *j*, corresponds to the number of asymmetric positions in the *j*^*th*^ mutant structure. The *AO*^*random*^ value of position *i* corresponds to the number of random sequences in which that position is considered an asymmetric position (i.e., showing a value of 1 at the position). *p*_*AO*_ is estimated as the probability that *AO*^*random*^ is higher than *AO(i)* using Eq. 1 and 2000 random sets.

Eq. 1 $${p}_{AO}(i)= prob\left[{AO}^{random}\right](i)> AO(i)=\frac{n\left\{{AO}^{random}(i)> AO(i)\right\}}{n_{simu}}$$

where [*n {AO*^*random*^*}(i) > AO(i)*] is the number of simulations in which *AO*^*random*^*(i)* is higher than *AO(i)*, and *n*_*simu*_ is the total number of simulations.

An asymmetric position was considered statistically overrepresented if its *p*_*AO*_ was smaller than a threshold of 0.0005, as determined using the Bonferroni adjustment to consider multiple tests (0.05/96 positions).

### Study of the link between mutations and changes in asymmetry

#### Location of changes in asymmetry in mutant structures relative to wild-type structure

A change in asymmetry corresponds to a position that exhibits a difference in asymmetry status (asymmetric or symmetric) in both a given mutant structure and the three 3EBZ_mini_ structures. Two types of changes in asymmetry were defined: asymmetry (or loss of asymmetry) occurs at a given position of a mutant structure if the position is asymmetric (or symmetric) in the mutant structure, whereas it is symmetric (or asymmetric) in the 3EBZ_mini_ structures. To locate the positions exhibiting changes in asymmetry in each mutant structure, we compared the asymmetry status in the mutant and 3EBZ_mini_ structures. To do so, we focused on only the 78 positions exhibiting the same status (asymmetric or symmetric) in the three 3EBZ_mini_ structures.

#### Quantification of the changes in asymmetry per mutant

For a given mutant, we computed the number of its five structures exhibiting changes in asymmetry for the 78 selected positions. This number was ranked from 0 to 5. A value of 0 for a position means that the position exhibits the same asymmetry status in the three 3EBZ_mini_ structures and the five structures of the mutant. In contrast, a position with a value of 5 means that the position exhibits a different asymmetry status in the three 3EBZ_mini_ structures and the five structures of the mutant. For all mutants, these data were summarized in a network that connects mutants with a position when at least one structure of the mutant exhibits a change in asymmetry relative to the wild-type structure at the position. The network was drawn using the *igraph* library of R software [57].

## Supplementary information

**Additional file 1.** Structural comparison between mutant structures and the three 3EBZ_mini_ structures.

**Additional file 2.** Summary of structural asymmetry in the three wild-type structures 3EBZ_mini_ and the 150 mutant structures.

**Additional file 3.** Flexibility of PR2 residues quantified using B-factor values.

**Additional file 4.** Exposure of PR2 residues quantified using ASA values.

**Additional file 5.** Quantification of structural asymmetry in the 150 structures of the PR2 mutants.

**Additional file 6.** Interaction between PR2 and three drugs (APV, DRV, and IDV).

**Additional file 7.** Residues involved in the interface of the wild-type PR2 dimer.

## Data Availability

The datasets used and/or analyzed during the current study are available from the corresponding author on reasonable request.

## Abbreviations

3D: Tridimensional
AO: Asymmetry occurrence
APV: Amprenavir
ASA: Accessible surface area
Cter: C-terminal end
DRV: Darunavir
FDA: Food and Drug Administration
HIV: Human immunodeficiency virus
HIV-1: Human immunodeficiency virus type 1
HIV-2: Human immunodeficiency virus type 2
HMM-SA: Hidden Markov Model - Structural Alphabet
IDV: Indinavir
LPV: Lopinavir
NFV: Nelfinavir
Nter: N-terminal end
OR_asym_: Over-represented asymmetric
PDB: Protein Data Bank
PI: Protease inhibitor
PME: Particle Mesh Ewald
PR1: HIV-1 protease
PR2: HIV-2 protease
RMSD: Root mean square deviation
RMSDaa: RMSD computed using all common atoms
SQV: Saquinavir
