# Supplementary material for: Impacts of drug resistance mutations on the structural asymmetry of the HIV-2 protease
Source: BMC Mol Cell Biol. 2020 Jun 23;21:46. doi: 10.1186/s12860-020-00290-1 (PMC7310402; doi:10.1186/s12860-020-00290-1)
Supplement: Supplementary file 1 — Additional file 1. Structural comparison between mutant structures and the three 3EBZmini structures. [file 12860_2020_290_MOESM1_ESM.pdf]

Additional file 1 —

A)

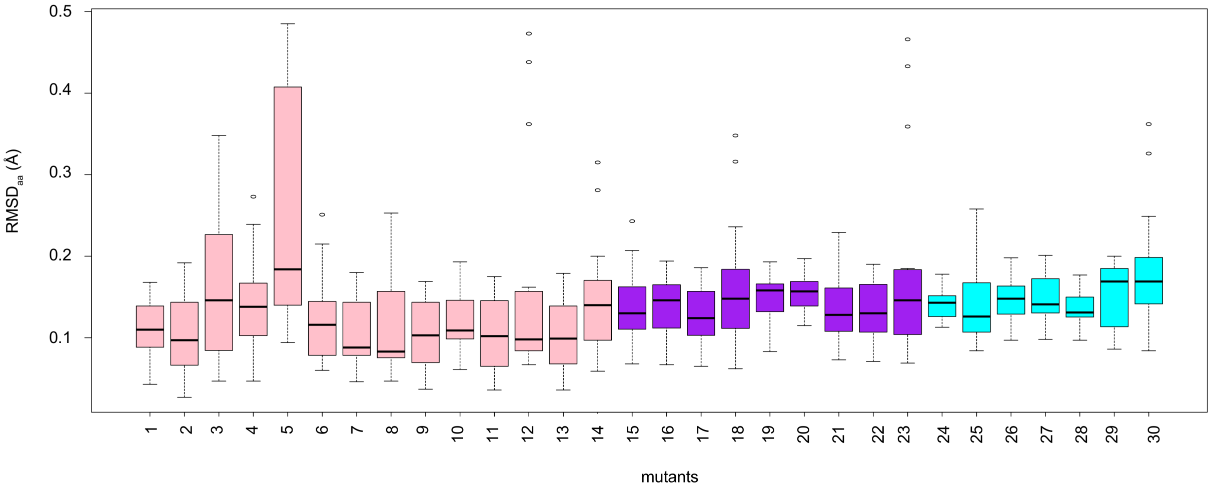

B)

	Mutant types			p-value
	single	double	triple	
Average $RMSD_{aa}$ (Å)	0.13	0.16	0.15	Kruskal-Wallis test: 6.10^{-7}
Standard deviation of $RMSD_{aa}$ (Å)	0.08	0.08	0.04	Bartlett test: 2.10^{-11}

C)

Comparisons of mutant types	Average *$RMSD_{AA}$* values comparison (Wilcoxon test pvalue)	SD *$RMSD_{AA}$* values comparison (F test pvalue)
Single versus double	4.10^{-05} (*)	0.7
Single versus triple	2.10^{-05} (*)	1.10^{-11} (*)
double versus triple	1	4.10^{-10} (*)

Figure S1 : (A) Distribution of the $RMSD_{aa}$ (Å) computed between the five mutant structures and the three $3EBZ_{mini}$ structures. Distributions of $RMSD_{aa}$ (Å) value for single, double, and triple mutants are colored in pink, purple, and cyan, respectively. The mutations included in each mutant, defined by their number, were listed in Figure 1A. Boxplot graphic displays the distribution of $RMSD_{aa}$ values for each mutant. The colored box indicates ``hinges'': the bottom and top limits correspond to the first and third quartiles of $RMSD_{aa}$ values. The central segment presents the median value. The bottom and top segments (``the whiskers'') are defined as 1.5 times the inter-quartile range. They correspond to the smallest and largest observations that falls within a distance of 1.5 times the box size from the nearest hinge. Circle points correspond to values that are outside the whiskers and they are considered as outliers. (B) Average value of $RMSD_{aa}$ (Å) for single, double, and triple mutants. (C) P-value of different tests that used to pairwise compare the average $RMSD_{aa}$ and its variability for single, double, and triple mutants. Each p-value was corrected using Bonferroni adjustment. (*) indicates significant p-value.
