# Supplementary material for: Impacts of drug resistance mutations on the structural asymmetry of the HIV-2 protease
Source: BMC Mol Cell Biol. 2020 Jun 23;21:46. doi: 10.1186/s12860-020-00290-1 (PMC7310402; doi:10.1186/s12860-020-00290-1)
Supplement: Supplementary file 2 — Additional file 2. Summary of structural asymmetry in the three wild-type structures 3EBZmini and the 150 mutant structures. [file 12860_2020_290_MOESM2_ESM.pdf]

Additional file 2 —

		Nter	R1	Fulcrum	Catalytic	R2	Elbow	Flaps	Cantilever	R3	Wall	R4	Helix	Cter
**Asymmetry in the three $3EBZ_{mini}$ wild-type structures**														
51 common symmetric positions	3, 6-11, 13, 14, 16, 17, 20, 24-26, 28-31, 34-36, 38, 39, 43, 45, 48, 49, 52-55, 57, 61-63, 65, 66, 68, 69, 71, 73, 76, 78, 81, 82, 84, 87, 94-96	1	4	7	6	4	2	9	9	2	2	1	3	1
27 common asymmetric positions	4, 5, 12, 18, 19, 21, 33, 40-42, 50, 51, 58-60, 64, 74, 75, 77, 83, 88, 89, 91-93, 97, 98	2	0	4	0	1	3	3	5	2	0	0	5	2
**Asymmetry in the 150 mutant structures**														
25 common symmetric positions	7, 9, 10, 13, 16, 17, 20, 28, 30, 35, 45, 52, 53, 55, 61, 66, 68, 69, 71, 73, 81, 82, 87, 94, 95	0	2	5	2	1	0	4	6	0	2	0	3	0
36 Or_{asym} positions	4, 5, 12, 15, 18, 19, 21, 23, 32, 33, 37, 42, 44, 47, 50, 51, 58-60, 64, 67, 74, 75, 77, 79, 80, 83, 86, 88-93, 97, 98	2	0	6	0	2	2	5	6	2	2	1	6	2
**Comparison of the asymmetry in the three $3EBZ_{mini}$ and the 150 mutant structures**														
25 positions are OR_{asym} in the mutant set and asymmetric in the three $3EBZ_{mini}$ structures	4, 5, 12, 18, 19, 21, 33, 42, 50, 51, 58-60, 64, 74, 75, 77, 83, 88, 89, 91-93, 97, 98	2	0	4	0	1	1	3	5	1	1	0	5	2
11 are Or_{asym} positions in the mutant set and are not asymmetric in the three $3EBZ_{mini}$ structures	15, 23, 32, 37, 44, 47, 67, 79, 80, 86, 90	0	0	2	0	1	1	2	1	1	1	1	1	0
2 are asymmetric in the three $3EBZ_{mini}$ structures and not over-represented in the mutant set	40 and 41	0	0	0	0	0	2	0	0	0	0	0	0	0

Figure S2: Summary of structural asymmetry in the three wild-type structures $3EBZ_{mini}$ and the 150 mutant structures. OR_{asym}: over-represented asymmetric positions.
