# Supplementary figures and images for: Impacts of drug resistance mutations on the structural asymmetry of the HIV-2 protease
Source: BMC Mol Cell Biol. 2020 Jun 23;21:46. doi: 10.1186/s12860-020-00290-1 (PMC7310402; doi:10.1186/s12860-020-00290-1)

### Additional file 3 —

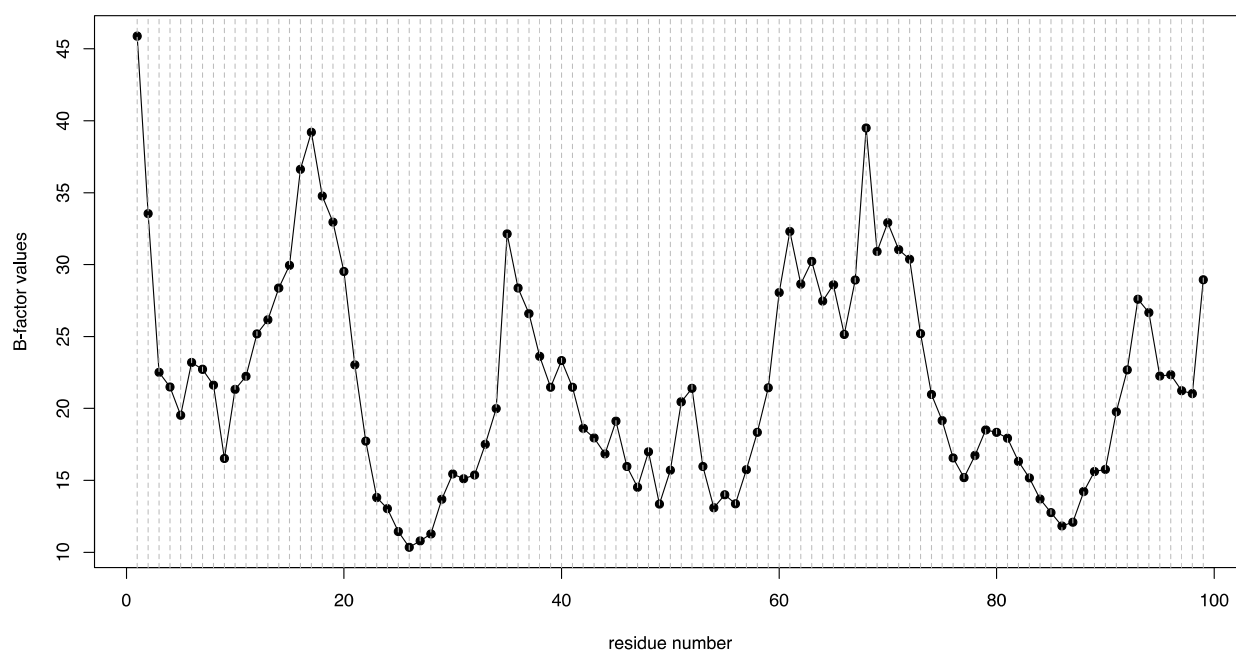

Figure S3: Flexibility of PR2 residues quantified using B-factor values.

Supplement: Supplementary file 3 — Additional file 3. Flexibility of PR2 residues quantified using B-factor values. [file 12860_2020_290_MOESM3_ESM.pdf]

## Additional file 4 —

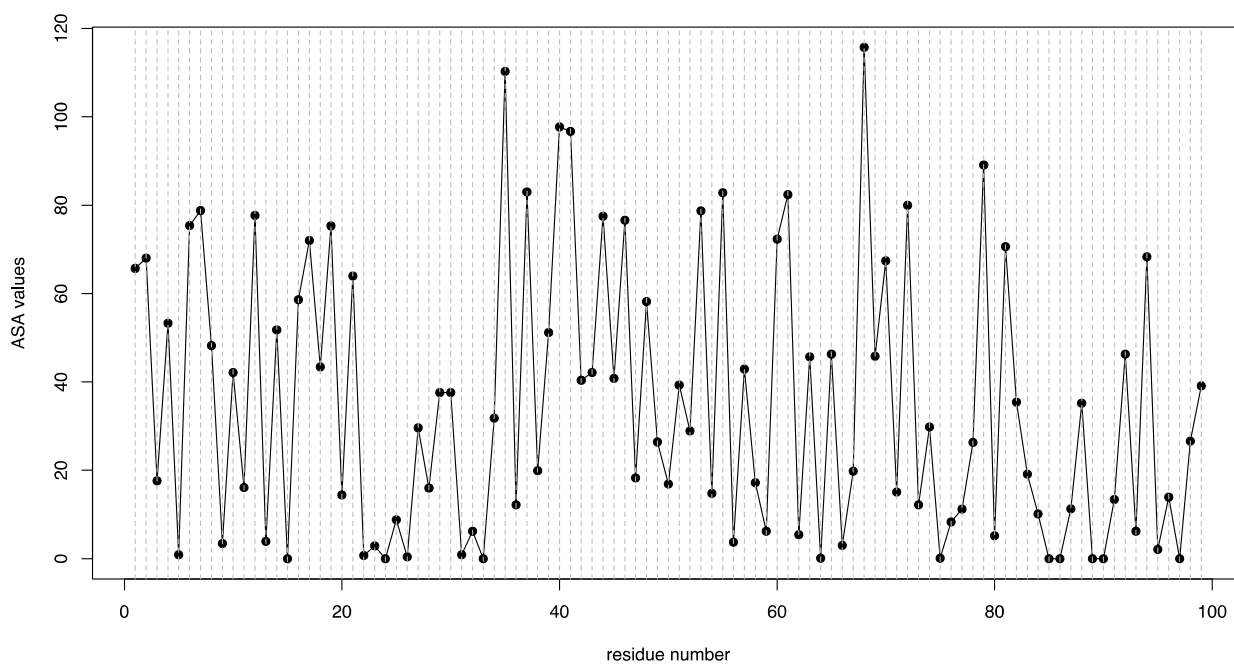

Figure S4: Exposure of PR2 residues quantified using ASA values (in  $\text{\AA}^2$ ) .

Supplement: Supplementary file 4 — Additional file 4. Exposure of PR2 residues quantified using ASA values. [file 12860_2020_290_MOESM4_ESM.pdf]
