# Supplementary material for: Impacts of drug resistance mutations on the structural asymmetry of the HIV-2 protease
Source: BMC Mol Cell Biol. 2020 Jun 23;21:46. doi: 10.1186/s12860-020-00290-1 (PMC7310402; doi:10.1186/s12860-020-00290-1)
Supplement: Supplementary file 5 — Additional file 5. Quantification of structural asymmetry in the 150 structures of the PR2 mutants. [file 12860_2020_290_MOESM5_ESM.pdf]

Additional file 5 —

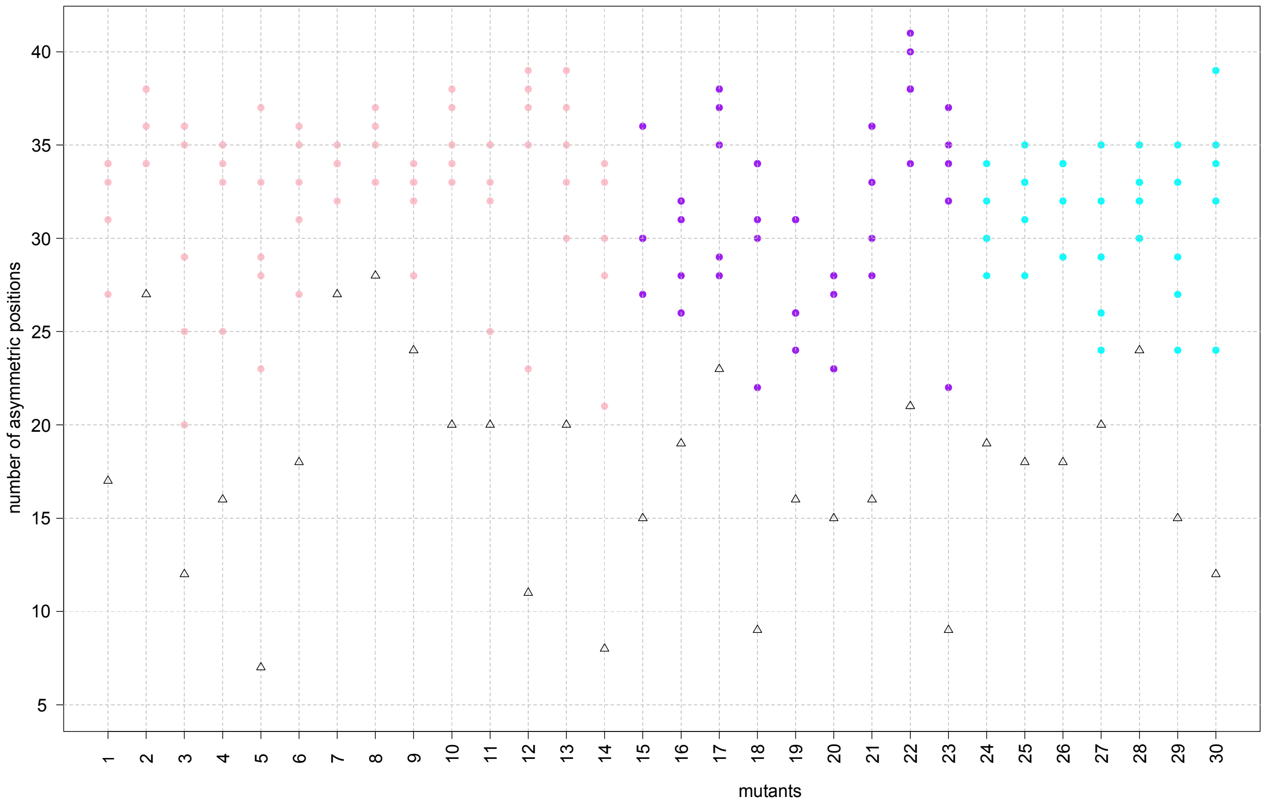

Figure S5: Quantification of structural asymmetry in the 150 structures of the PR2 mutants. Single double, and triple mutants are colored in pink, purple, and cyan, respectively. Triangles indicate the number of common asymmetric positions amongst the five structures of a given mutant.
