# Supplementary material for: Impacts of drug resistance mutations on the structural asymmetry of the HIV-2 protease
Source: BMC Mol Cell Biol. 2020 Jun 23;21:46. doi: 10.1186/s12860-020-00290-1 (PMC7310402; doi:10.1186/s12860-020-00290-1)
Supplement: Supplementary file 6 — Additional file 6. Interaction between PR2 and three drugs (APV, DRV, and IDV). [file 12860_2020_290_MOESM6_ESM.pdf]

Additional file 6 —

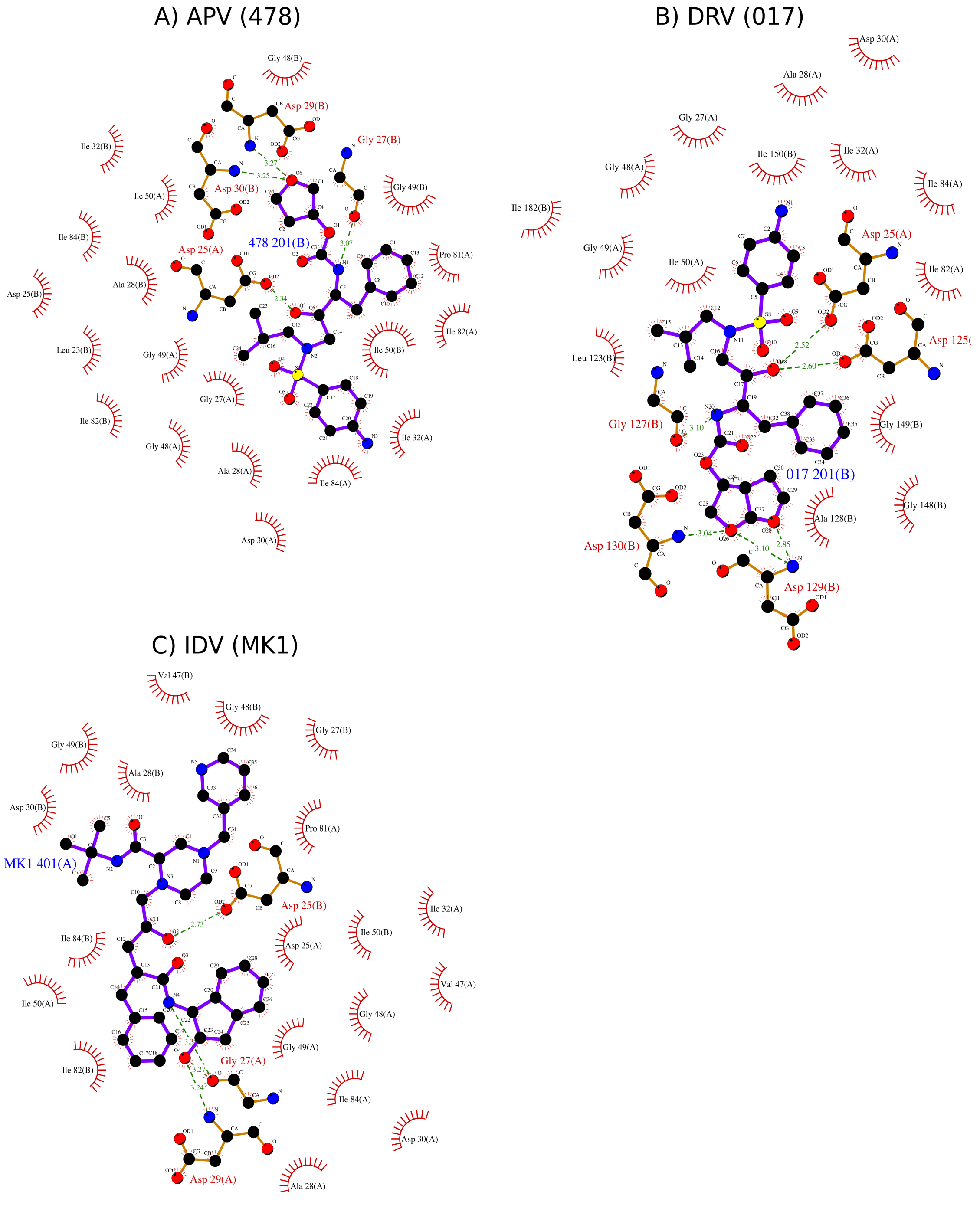

Figure S6: Description of interactions between PR2 and three FDA-approved PIs: APV (A), DRV (B), and IDV (C) using LIGPLOT diagrams. These figures were generated using LigPlot software [1] using PDB files 3S45 (A), 3EBZ (B), and 1HSH (C). Hydrogen bonds are shown as green dotted lines labelled with the length of the bond in Å. Protein residues that make one or more non-bonded contacts to the ligand are shown as dark red "eyelashes".

[1] Wallace, A.C., Laskowski, R.A., Thornton, J.M.: Ligplot: a program to generate schematic diagrams of protein-ligand interactions. Protein Eng. 8, 127–134 (1996)
