# Supplementary material for: Impacts of drug resistance mutations on the structural asymmetry of the HIV-2 protease
Source: BMC Mol Cell Biol. 2020 Jun 23;21:46. doi: 10.1186/s12860-020-00290-1 (PMC7310402; doi:10.1186/s12860-020-00290-1)
Supplement: Supplementary file 7 — Additional file 7. Residues involved in the interface of the wild-type PR2 dimer. [file 12860_2020_290_MOESM7_ESM.pdf]

Additional file 7 —

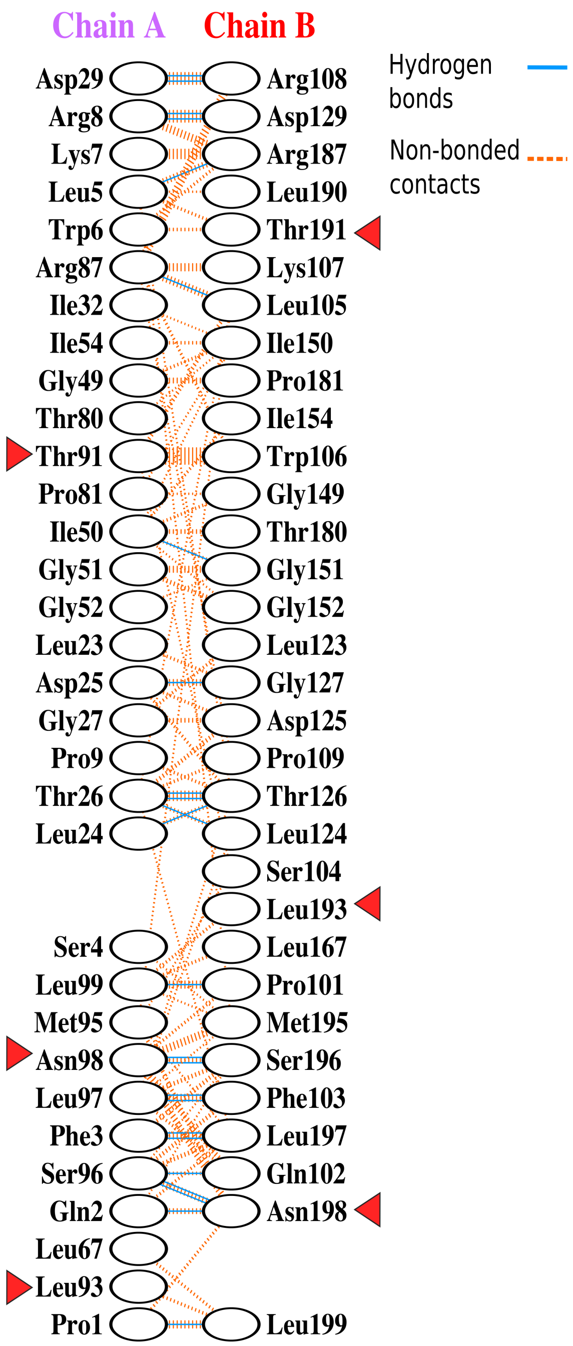

Figure S7: Residues involved in the interface of the wild-type PR2 dimer. Diagram presenting interactions between residues of the two chains of wild-type PR2. Red triangles indicate interface residues highlighted in this work.
